# Safety and oncologic outcomes of total laparoscopic versus abdominal hysterectomy following diagnostic conization for adenocarcinoma in situ and stage IA1 cervical cancer: a multicenter retrospective study

**DOI:** 10.1007/s10147-026-02971-x

**Published:** 2026-01-30

**Authors:** Yoshitaka Kaido, Masahiro Kagabu, Yohei Chiba, Sho Sato, Eriko Takatori, Takayuki Nagasawa, Tadahiro Shoji, Manami Sakurai, Tatsuhiko Shigeto, Kenichi Makino, Tsuyoshi Ohta, Shogo Shigeta, Tomoyuki Nagai, Michiko Kaiho-Sakuma, Hidemichi Watari, Satoru Nagase, Hideki Tokunaga, Tsukasa Baba, Yoshihito Yokoyama

**Affiliations:** 1 Department of Obstetrics and Gynecology, Iwate Medical University School of Medicine, 2-1-1, Idaidori, Yahaba, Iwate 028-3695 Japan; 2 Department of Obstetrics and Gynecology, Faculty of Medicine, Hokkaido University, Sapporo, Japan; 3 Department of Obstetrics and Gynecology, Faculty of Medicine, Hirosaki University, Hirosaki, Japan; 4 Department of Obstetrics and Gynecology, Faculty of Medicine, Akita University, Akita, Japan; 5 Department of Obstetrics and Gynecology, Faculty of Medicine, Yamagata University, Yamagata, Japan; 6 Department of Obstetrics and Gynecology, School of Medicine, Tohoku University, Sendai, Japan; 7 Department of Gynecology, Miyagi Cancer Center, Natori, Miyagi Japan; 8 Division of Obstetrics and Gynecology, Faculty of Medicine, Tohoku Medical and Pharmaceutical University, Sendai, Japan

**Keywords:** Total laparoscopic hysterectomy, Adenocarcinoma in situ, Stage IA1 cervical cancer, Diagnostic conization, Minimally invasive surgery, Surgical outcomes

## Abstract

**Background:**

While simple hysterectomy is the standard treatment for adenocarcinoma in situ (AIS) and stage IA1 cervical cancer, minimally invasive surgery has been increasingly adopted. However, evidence on the safety and efficacy of total laparoscopic hysterectomy (TLH) for these conditions remains limited. We compared the safety and efficacy of TLH and total abdominal hysterectomy (TAH) in patients undergoing simple hysterectomy following diagnostic conization.

**Methods:**

We conducted a multicenter retrospective study of 196 patients with cervical intraepithelial neoplasia grade 3 (CIN3), AIS, or stage IA1 cervical cancer who underwent simple hysterectomy following diagnostic conization. Patients were divided into TLH and TAH groups, and intraoperative and postoperative complications and oncologic outcomes were compared.

**Results:**

Operative time was significantly longer in the TLH group, whereas intraoperative blood loss was lower and postoperative hospital stay shorter. No significant difference was noted in severe complication rates, although their patterns varied between groups. Recurrence occurred in one patient (0.5%), a case of CIN3 at the vaginal cuff in the stage IA1 TAH group. No recurrences were observed in patients with AIS or stage IA1 disease in the TLH group.

**Conclusions:**

TLH following diagnostic conization for AIS or stage IA1 cervical cancer demonstrated oncologic efficacy comparable to TAH, with a favorable safety profile. TLH may be a reasonable treatment option for carefully selected patients.

**Supplementary Information:**

The online version contains supplementary material available at 10.1007/s10147-026-02971-x.

## Introduction

Cervical cancer ranks as the fourth most common cancer among women globally, with approximately 661,021 new cases and 348,189 deaths reported in 2022 [1]. Minimally invasive surgery (MIS), including laparoscopic and robot-assisted approaches, has been increasingly adopted for the surgical management of gynecologic malignancies. According to the annual report of the Japan Society of Gynecologic Oncology (JSGO), the proportion of MIS among hysterectomy cases for stage IA1 cervical cancer increased from 40.5% in 2018 to 53.4% in 2021, with indications for MIS continuing to expand annually [2].

Among MIS techniques, total laparoscopic hysterectomy (TLH) without lymph node dissection is considered the standard treatment for IA1 disease without lymphovascular space invasion (LVSI) or adenocarcinoma in situ (AIS). TLH is an established surgical procedure for hysterectomy in benign uterine conditions, and previous reports indicate that TLH offers advantages over total abdominal hysterectomy (TAH), including shorter postoperative hospital stay and reduced blood loss [3]. However, robust evidence comparing TLH and TAH as surgical approaches for early-stage cervical malignancies remains limited. Notably, the LACC trial for stage IA2 to IB1 cervical cancer demonstrated that the efficacy and safety of laparoscopic radical hysterectomy were inferior to open surgery, prompting careful reconsideration of its indications [4]. Nevertheless, a gap persists between clinical guidelines and actual practice, complicating surgical decision-making in daily clinical care.

Considering these factors, this multicenter retrospective study aimed to evaluate the safety and oncologic outcomes of TLH following diagnostic conization for early-stage cervical cancer and assess its appropriateness as a treatment option compared with TAH.

## Patients and methods

### Study design

This retrospective study included patients diagnosed between January 2011 and December 2020 with stage IA1 cervical cancer without LVSI, as defined by the FIGO 2018 classification, or with AIS or CIN3, who underwent simple hysterectomy without lymphadenectomy via either an open or laparoscopic approach as definitive treatment.

The safety of laparoscopic surgery was evaluated in patients with CIN3, AIS, and stage IA1 disease using operative blood loss, operative time, postoperative hospital stay, and the incidence of intraoperative and postoperative complications as parameters. Efficacy was assessed in patients with AIS and stage IA1 disease based on recurrence rates. In addition, the presence of residual lesions in the resected uterus was examined. This study was approved by the Institutional Review Board (IRB) of Iwate Medical University School of Medicine (approval number: MH2022-038; approval date: May 29, 2023).

### Participating institutions

Eligible cases were collected from 7 institutions participating in the Tohoku Gynecologic Cancer Unit: Iwate Medical University, Hokkaido University, Hirosaki University, Akita University, Tohoku University, Yamagata University, and Miyagi Cancer Center. A total of 196 patients were included in the final analysis. All cases received approval from the respective IRBs of the participating centers.

### Surgical procedures

Disease staging based on the FIGO 2018 classification was determined from histopathological findings of diagnostic conization. Both NCCN and JSGO guidelines recommend simple hysterectomy without lymphadenectomy for AIS and stage IA1 cervical cancer without LVSI [5, 6]. The choice between open and laparoscopic surgery was made at each institution’s discretion. In this study, adnexal removal and the use of a uterine manipulator during laparoscopic surgery were not included as analytic variables.

### Statistical analysis

All statistical analyses were performed using EZR (version 1.68; Saitama Medical Center, Jichi Medical University, Saitama, Japan), a graphical user interface for R (version 2.9–1; R Foundation for Statistical Computing, Vienna, Austria). EZR is a modified R Commander that integrates statistical functions widely applied in biostatistics [7].

Fisher’s exact test was used for categorical variables in 2 × 2 tables, while the chi-square test was applied for larger contingency tables, and the Mann–Whitney U test was used for continuous variables. The significance level was set at *P* < 0.05.

## Results

### Patient characteristics

Among the 196 patients included in this study, 72 had CIN3 (32 TAH, 40 TLH), 53 had AIS (29 TAH, 24 TLH), and 71 had stage IA1 cervical cancer (54 TAH, 17 TLH). Overall patient characteristics are summarized in Table 1. Median age showed no statistically significant difference between the TLH and TAH groups (*P* = 0.49). However, a significant difference was noted in the distribution of pathological diagnoses between the groups (*P* < 0.01), with fewer stage IA1 cases in the TLH group. Median follow-up duration was significantly shorter in the TLH group than in the TAH group (median, 34.5 vs. 55 months; *P* = 0.04).

**Table 1:** Baseline patient characteristics

	TAH (*n* = 115)	TLH (*n* = 81)	*P*-value
Median age, years (range)	45 (31–73)	45 (29–77)	0.49^a^
Clinical stage			< 0.01^b^
IA1	54	17	
AIS	29	24	
CIN3	32	40	
Median follow-up period, months (range)	55 (0–132)	34.5 (0–145)	0.004^a^

TAH, total abdominal hysterectomy; TLH, total laparoscopic hysterectomy; AIS, adenocarcinoma in situ, CIN3, cervical intraepithelial neoplasia grade 3

^a^Mann–Whitney U test

^b^Chi-square test

### Safety outcomes

Operative time was significantly longer in the TLH group (median, 181 vs. 117 min; *P* < 0.001). In contrast, estimated blood loss was significantly lower (median, 19 vs. 76 mL; *P* < 0.001), and postoperative hospital stay was significantly shorter (median, 5 vs. 8 days; *P* < 0.0001) in the TLH group.

Complications occurred in five patients (4.3%) in the TAH group and 1 patient (1.2%) in the TLH group, with no significant difference in incidence (*P* = 0.40) (Table 2).

**Table 2:** Safety outcomes of total laparoscopic and total abdominal hysterectomies

	TAH (*n* = 115)	TLH (*n* = 81)	*P*-value
Median operating time, minutes (range)	117 (30–327)	181 (88–340)	< 0.01^a^
Median estimated blood loss, mL (range)	76 (5–911)	19 (0–1860)	< 0.01^a^
Median postoperative hospital stay, days (range)	8 (5–24)	5 (3–17)	< 0.01^a^
Number of complications, n (%)	5 (4.3%)	1 (1.2%)	0.40^b^

TAH, total abdominal hysterectomy; TLH, total laparoscopic hysterectomy

^a^Mann–Whitney U test

^b^Fisher’s exact test

Postoperative complications in the TAH group included one case of bowel obstruction, two cases of vaginal vault hematoma, and two cases of vaginal vault abscess. In the TLH group, one patient needed a ureteral stent due to ureteral stricture. All six cases were managed conservatively without requiring reoperation or other invasive procedures, and all patients recovered well.

### Oncologic outcomes

Recurrence was defined as histopathological confirmation of carcinoma in situ, a more advanced lesion, or radiological evidence of recurrence.

Among the 124 patients with AIS (*n* = 53) or stage IA1 cervical cancer (*n* = 71), only 1 recurrence was observed, occurring in the TAH group with stage IA1 disease. No recurrences were detected in patients with AIS, and no pelvic, nodal, or distant recurrences were observed in any patient.

### Stage IA1 disease

In the TAH group, 1 patient developed CIN3 recurrence at the vaginal cuff, underwent laser ablation, and remains disease-free. No significant differences were identified between the TAH and TLH groups in median age (*P* = 0.86), follow-up duration (*P* = 0.87), histologic type (*P* = 0.53), or recurrence rate (*P* = 1.0) (Table 3).

**Table 3:** Efficacy of total laparoscopic and total abdominal hysterectomy in patients with Stage IA1

	TAH (*n* = 54)	TLH (*n* = 17)	*P*-value
Median age, years (range)	45 (31–73)	40 (31–65)	0.86^a^
Number of histological type			0.53^b^
SCC	39 (72.2%)	14 (82.4%)	
Adenocarcinoma	15 (27.8%)	3 (17.6%)	
Median follow-up period, months (range)	60.5 (0–128)	60 (2–145)	0.87^a^
Number of recurrence	1	0	1.0^a^
Number of deaths	0	0	1.0^a^

TAH, total abdominal hysterectomy; TLH, total laparoscopic hysterectomy; SCC, squamous cell carcinoma

^a^Mann–Whitney U test

^b^Fisher’s exact test

### AIS

No recurrence was observed. Median follow-up duration was significantly shorter in the laparoscopic group than in the open group (35 vs. 60 months; *P* = 0.037), with no significant differences in median age (*P* = 0.31) or recurrence rate (*P* = 1.0) (Table 4).

**Table 4:** Efficacy of total laparoscopic hysterectomy and total abdominal hysterectomy in patients with adenocarcinoma in situ

	TAH (*n* = 29)	TLH (*n* = 24)	*P*-value
Median age, years (range)	44 (36–71)	42 (29–73)	0.31^a^
Median follow-up period, months (range)	60 (1–117)	34.5 (4–95)	0.037^a^
Number of recurrence	0	0	1.0^b^
Number of deaths	0	0	1.0^b^

TAH, total abdominal hysterectomy; TLH, total laparoscopic hysterectomy

^a^Mann–Whitney U test

^b^Fisher’s exact test

### Residual disease in the resected uterus

Among 53 patients with AIS who underwent hysterectomy, residual AIS lesions were identified in 2 of 32 patients (6.3%) with negative conization margins.

Among 71 patients with stage IA1 cervical cancer who underwent hysterectomy, residual lesions were identified in 8 of 44 patients (18%) with negative conization margins (Online Resource 1).

## Discussion

Our multicenter analysis of nearly 200 patients is one of the largest cohorts directly comparing TLH and TAH following diagnostic conization for AIS and stage IA1 cervical cancer. In contrast to earlier single-institution studies with limited sample sizes, this study provides practice-oriented evidence across diverse clinical settings, thereby enhancing the external validity of TLH as a treatment option.

We first examined safety. Overall complication rates showed no significant difference between the TLH and TAH groups, although the patterns of complications differed. In the TAH group, five patients experienced infection- or adhesion-related complications, including one bowel obstruction, two vaginal vault hematomas, and two vaginal vault abscesses. In contrast, one case of ureteral stricture was observed in the TLH group. According to a Cochrane review, TLH tends to carry a higher risk of ureteral injury than TAH in benign gynecologic conditions, while TLH generally shows a lower incidence of wound infection and postoperative fever; however, the evidence on pelvic infection remains inconclusive [8]. Walsh et al. reported in a meta-analysis of randomized controlled trials (RCTs) for benign gynecologic conditions that TAH tends to have a higher risk of postoperative hematoma than TLH [9]. These findings indicate that the complication profiles associated with simple hysterectomy after conization remain consistent with those observed in benign conditions. Several retrospective studies (each with approximately 30 cases) reporting TLH after conization have emphasized vigilance regarding urinary tract injury. In the present study, ureteral injury occurred in 1 of 81 TLH cases (1.2%). Similarly, Hoshino et al. reported ureteral injury in 1 of 32 cases (3.1%) [10], and Phongnarisorn et al. reported it in 1 of 26 cases (3.8%) [11]. In contrast, Léonard et al. reported a ureteral injury rate of 0.3% for TLH performed for benign conditions [12], while Chang et al. reported a rate of 0.13% [13]. These data indicate that TLH after conization may increase the risk of ureteral injury due to postoperative inflammation, retroperitoneal fibrosis, and altered pelvic anatomy. Therefore, heightened perioperative vigilance is necessary. Overall, TLH following diagnostic conization appears technically feasible and comparable in safety to TAH; however, comprehensive preoperative risk assessment and intraoperative precautions, particularly to prevent ureteral injury, are essential when considering patient safety.

Next, we examined the efficacy. Current guidelines for early-stage cervical cancer, including those from the NCCN and JSGO, recommend simple hysterectomy for AIS and stage IA1 cervical cancer without LVSI [5, 6]. The 2024 Guidelines of the Japan Society of Gynecologic Endoscopy recognize laparoscopic surgery as a viable alternative to open surgery; however, the evidence level remains Grade C [14], underscoring the need for further validation. In this study, recurrence was observed in only 1 case (0.5%) out of 124 patients with AIS or stage IA1. This specific case involved a recurrence of CIN3 at the vaginal stump in the TAH group with stage IA1 disease. No recurrences were observed in the TLH group for either AIS or stage IA1, and no lymph node or distant metastases were identified. Thus, both groups demonstrated excellent oncologic outcomes. By disease stage, AIS cases showed no recurrences regardless of surgical approach, and IA1 cases had recurrence rates of 0% in the TLH group and one case in the TAH group. These findings support TLH as a valid treatment option, demonstrating oncologic efficacy equivalent to TAH following diagnostic conization. Furthermore, the 65th Annual Report of the JSOG reported a 5-year overall survival rate of 99.4% for stage IA1 disease [15]. All patients in this study remained alive throughout the follow-up period. These results are highly reliable due to the median follow-up duration of 60 months and the strict recurrence definition, which included carcinoma in situ.

Recent studies have also examined the appropriateness of minimally invasive surgery for microinvasive cervical cancer. Togami et al. reported no difference in oncologic outcomes between laparoscopic and open hysterectomy for stage IA1 cervical cancer; however, their cohort included cases with LVSI, and surgical procedures more extensive than simple hysterectomy—such as modified radical hysterectomy and sentinel node navigation surgery (SNNS)—were mixed within the analysis [16]. Similarly, Hayek et al., using the National Cancer Database (NCDB), demonstrated comparable survival between minimally invasive and open surgery for stage IA1–IA2 disease, but their study also combined simple and radical hysterectomy procedures and did not account for factors such as prior diagnostic conization, perioperative outcomes (including blood loss and complications), or recurrence patterns [17]. In contrast, our study focused exclusively on a homogeneous cohort of patients with AIS or stage IA1 cervical cancer without LVSI who underwent simple hysterectomy following diagnostic conization, thereby closely reflecting current guideline-based clinical practice. These distinctions allow our findings to complement the existing literature while providing more targeted, real-world evidence regarding the safety and oncologic validity of TLH performed after diagnostic conization.

We also discussed the role of less radical surgery, which has gained increasing attention in recent years. In gynecologic oncology, interest in both minimally invasive and less radical surgical strategies is growing. The SHAPE and ConCerv trials investigated whether conization or simple hysterectomy is noninferior to radical hysterectomy in patients with stage IA2–IB1 cervical cancer, where radical hysterectomy has traditionally been the standard, and reported favorable outcomes [18, 19]. However, these studies involved a different population than ours (AIS and stage IA1 without LVSI) and required pelvic lymphadenectomy. Furthermore, both trials were designed to determine whether prognosis remains comparable following fewer radical procedures. Although our study was retrospective rather than prospective, it compared surgical approaches to total hysterectomy (laparoscopic versus open). The LACC trial, an extensive comparative study of radical hysterectomy for cervical cancer (laparoscopic versus open), failed to demonstrate the noninferiority of laparoscopic surgery [4]. Therefore, despite its retrospective design, this study provides valuable insights into the feasibility and oncologic safety of laparoscopic hysterectomy for early-stage disease.

This study has several limitations. The surgical approach was determined by each institution and surgeon, potentially introducing selection bias. Although this was not a randomized study, the data nonetheless represent a decade of evolving clinical practice. Many TLH cases occurred during the early phase of MIS adoption, yet no recurrences were observed, reinforcing oncologic safety. However, the participation of multiple institutions and surgeons provides outcomes reflecting diverse skill levels and real-world conditions, thereby improving external validity.

Additionally, this was a retrospective study, inherently limited compared with prospective RCTs. However, in AIS and stage IA1 cervical cancer, where prognosis is highly favorable, RCTs face both statistical and ethical challenges. Moreover, because MIS is now widely used, randomizing large numbers of patients to TAH is impractical. Therefore, multicenter retrospective analyses such as ours may yield valuable practice-oriented evidence where RCTs are challenging to perform.

Despite these limitations, our findings provide real-world evidence supporting TLH as a feasible and safe alternative to TAH in appropriately selected patients.

This study demonstrates that performing TLH following diagnostic conization results in oncologic outcomes and safety that are comparable to those of TAH. Therefore, TLH following diagnostic conization for AIS and stage IA1 cervical cancer without LVSI appears to be a safe and effective treatment option, supporting its adoption as a minimally invasive alternative for appropriately selected patients.

## Supplementary Information

Below is the link to the electronic supplementary material.

Supplementary file1 (DOCX 25 kb) Online Resource 1: Residual lesions in the excised uterus among patients with negative conization margins (Docx)

## Data Availability

All data generated or analyzed during this study are included in this published article and its supplementary information files. Additional datasets or analyses not presented in the manuscript are available from the corresponding author upon reasonable request.
