# Supplementary material for: Safety and oncologic outcomes of total laparoscopic versus abdominal hysterectomy following diagnostic conization for adenocarcinoma in situ and stage IA1 cervical cancer: a multicenter retrospective study
Source: Int J Clin Oncol. 2026 Jan 30;31(3):558–63. doi: 10.1007/s10147-026-02971-x (PMC12932381; doi:10.1007/s10147-026-02971-x)
Supplement: Supplementary file 1 — Supplementary file1 (DOCX 25 kb) Online Resource 1: Residual lesions in the excised uterus among patients with negative conization margins (Docx) [file 10147_2026_2971_MOESM1_ESM.docx]

**Article Title:** Safety and Oncologic Outcomes of Total Laparoscopic versus Abdominal Hysterectomy Following Diagnostic Conization for Adenocarcinoma in situ and Stage IA1 Cervical Cancer: A Multicenter Retrospective Study

**Journal:** *International Journal of Clinical Oncology (IJCO)*

**Authors:** Yoshitaka Kaido^1^, Masahiro Kagabu^1^, Yohei Chiba^1^, Sho Sato^1^, Eriko Takatori^1^, Takayuki Nagasawa^1^, Tadahiro Shoji^1^, Manami Sakurai^2^, Tatsuhiko Shigeto^3^, Kenichi Makino^4^, Tsuyoshi Ohta^5^, Shogo Shigeta^6^, Tomoyuki Nagai^7^, Michiko Kaiho-Sakuma^7^, Hidemichi Watari^2^, Satoru Nagase^5^, Hideki Tokunaga^8^, Tsukasa Baba^1^, Yoshihito Yokoyama^3^

^1^Department of Obstetrics and Gynecology, Iwate Medical University School of Medicine

^2^Department of Obstetrics and Gynecology, Faculty of Medicine, Hokkaido University, Sapporo, Japan

^3^ Department of Obstetrics and Gynecology, Faculty of Medicine, Hirosaki University, Hirosaki, Japan

^4^ Department of Obstetrics and Gynecology, Faculty of Medicine, Akita University, Akita, Japan

^5^Department of Obstetrics and Gynecology, Faculty of Medicine, Yamagata University, Yamagata, Japan

^6^Department of Obstetrics and Gynecology, School of Medicine, Tohoku University, Sendai, Japan

^7^Department of Gynecology, Miyagi Cancer Center, Natori, Miyagi, Japan

^8^Division of Obstetrics and Gynecology, Faculty of Medicine, Tohoku Medical and Pharmaceutical University, Sendai, Japan

**Corresponding Author:** Masahiro Kagabu, Department of Obstetrics and Gynecology, Iwate Medical University School of Medicine, 2-1-1 Idaidori, Yahaba, Iwate 028-3695, Japan. Email: mkagabu@iwate-med.ac.jp

**Online Resource 1**

Residual lesions in the excised uterus in cases with negative margins

| Disease stage | Number of cases with residual lesions (%) |
| --- | --- |
| Stage IA1 | 8 (18%) |
| AIS | 2 (6.3%) |

AIS: adenocarcinoma in situ
